# Theranostic Potential of the iPSMA-Bombesin Radioligand in Patients with Metastatic Prostate Cancer: A Pilot Study

**DOI:** 10.3390/pharmaceutics16111358

**Published:** 2024-10-24

**Authors:** Sofía González-Rueda, Osvaldo García-Pérez, Myrna Luna-Gutiérrez, Blanca Ocampo-García, Clara Santos-Cuevas, Gerardo Ramírez-Nava, Joel Vargas-Ahumada, Erika Azorín-Vega, Guillermina Ferro-Flores, Laura Meléndez-Alafort

**Affiliations:** 1 Department of Nuclear Medicine, Instituto Nacional de Cancerología, Mexico City 14080, Mexico; 2 Department of Radioactive Materials, Instituto Nacional de Investigaciones Nucleares (ININ), Ocoyoacac 52750, Mexico; 3 Institute of Advanced Materials for Sustainable Manufacturing, School of Engineering and Sciences, Tecnológico de Monterrey, Mexico City 14380, Mexico; 4 Immunology and Molecular Oncology Diagnostics Unit, Veneto Institute of Oncology IOV-IRCCS, 35128 Padua, Italy

**Keywords:** PSMA, GRPR, prostate cancer, heterodimer radiotracer, SPECT, ^99m^Tc/^177^Lu

## Abstract

**Background/Objectives:** Prostate cancer (PC) represents the second most diagnosed form of cancer in men on a global scale. Despite the theranostic efficacy of prostate-specific membrane antigen (PSMA) radioligands, there is a spectrum of PC disease in which PSMA expression is low or absent. The gastrin-releasing peptide receptor (GRPR), also known as the bombesin type 2 receptor, has been identified as a target in both the early and advanced stages of PC. The objective of this study was to prepare and preclinically evaluate [^99m^Tc]Tc-iPSMA-Bombesin ([^99m^Tc]Tc-iPSMA-BN), estimate dosimetry in healthy subjects, and assess the diagnostic efficacy of the radiotracer in patients with metastatic PC, with the hypothesis of non-inferiority to one of the gold standards, [^18^F]-PSMA-1007. Moreover, the potential of [^99m^Tc]Tc-iPSMA-BN as a theranostic pair with [^177^Lu]Lu-iPSMA-BN was investigated. **Methods:** [^99m^Tc]Tc-iPSMA-BN was prepared under GMP conditions with radiochemical purities > 95%, showing specific recognition by PSMA and GRP receptors in prostate cancer cells and mice bearing PC tumors. Six healthy volunteers were enrolled, and [^99m^Tc]Tc-iPSMA-BN SPECT/CT imaging (740 MBq) was performed to estimate the dosimetry. The pilot clinical study included seven mCRPC and four mCSPC patients with prior androgen deprivation therapy. All patients had a recent [^18^F]-PSMA-PET/CT scan and were enrolled in this prospective study on their own signed behalf. Volumetric lesion target-to-background ratios (TBRs) were obtained from PET/CT and SPECT/CT images. **Results:** [^99m^Tc]Tc-iPSMA-BN effective radiation dose was 1.94 ± 0.39 mSv/740 MBq. A total of 178 lesions were detected via CT, 162 via [^18^F]-PSMA-1007 PET, and 155 via [^99m^Tc]Tc-iPSMA-BN SPECT. Three patients with mCRPC had higher TBR values on SPECT than on PET. [^99m^Tc]Tc-iPSMA-BN appears to have better lesion detection in patients with aggressive histologic transformation. Two-way ANOVA analysis revealed a significant difference in TBR values between patients with mCRPC and mCSPC (*p* < 0.05) but no difference between [^18^F]-PSMA-1007 and [^99m^Tc]Tc-iPSMA-BN (*p* > 0.05). In one patient, [^177^Lu]Lu-iPSMA-BN showed a high correlation with [^99m^Tc]Tc-iPSMA-BN for lesions that concentrated radioactivity. **Conclusions:** [^99m^Tc]Tc-iPSMA-BN SPECT/CT is a promising alternative not only for diagnostic purposes but also for broadening the spectrum of PC patients who may benefit from radionuclide theranostics. The results justify the development of a clinical trial involving a significant number of patients with PC.

## 1. Introduction

Prostate cancer (PC) is one of the most prevalent malignant neoplasms globally. It currently ranks second in terms of incidence and fifth in terms of mortality among males worldwide [1]. Given its high prevalence and mortality rates, PC represents a significant public health concern that necessitates prompt and effective diagnosis and treatment across all clinical stages. In accordance with the Surveillance, Epidemiology, and End Results (SEER) Program of the National Cancer Institute of the United States of America (2019), PC cases are classified as follows at the time of diagnosis: the majority (69%) are categorized as localized disease, while 13% are regional, and 8% are metastatic. A further 10% of cases are classified as indeterminate [2].

Diagnostic and therapeutic methods are assigned in accordance with the stage of the patient’s disease. This is based on the natural history of PC, which progresses from non-metastatic castration-sensitive (CSPC) to metastatic castration-sensitive (mCSPC). Additionally, there is an intermediate category of castration-resistant prostate cancer without metastasis (m0CRPC), which subsequently progresses to metastatic castration-resistant prostate cancer (mCRPC). Previously, patients with PC were staged with conventional imaging studies, usually consisting of a contrast-enhanced computed tomography (CT) scan of the thorax, abdomen, and pelvis, as well as a bone scan with [^99m^Tc]Tc-biphosphonates [3]. However, in recent years, numerous studies using a variety of methodologies, including clinical guidelines, medical society compendia, randomized clinical trials, and meta-analyses, have significantly influenced the repositioning of the new generation of medical imaging. In particular, these studies have highlighted the value of molecular imaging modalities such as positron emission tomography (PET) with CT, which can detect highly specific proteins expressed in the cell membrane of neoplastic prostate cells, for instance prostate-specific membrane antigen (PSMA). PSMA PET/CT has been shown to be particularly useful in advanced disease [4], biochemical recurrence [5], and, more recently, in localized stages prior to curative-intent therapy in high-risk patients [6]. Single-photon emission computed tomography (SPECT) is another imaging modality with similar diagnostic possibilities to PET/CT in providing accurate molecular imaging. In this regard, Vargas-Ahumada et al. recently analyzed the diagnostic performance of [^99m^Tc]Tc-iPSMA SPECT/CT in the staging of patients with unfavorable intermediate-, high-, and very high-risk prostate cancer compared to [^18^F]-PSMA-1007 PET/CT, in which they performed a volumetric analysis of the lesions in all patients, without finding statistically significant differences between the two imaging modalities [7].

Nevertheless, there is a spectrum of PC disease in which there is a lower expression of PSMA or even its complete absence. This may be attributed to many factors but can be broadly classified into two categories. The first one encompasses low-grade, non-invasive PC with a less aggressive disease spectrum. These neoplasms may not yet exhibit some of the hallmarks characteristic of most PSMA-avid tumors, including angiogenesis, genomic instability, and invasiveness [8]. The second category represents the opposite end of the spectrum, which includes neoplasms with a lower degree of cellular differentiation (Gleason score 9–10), high aggressiveness, capacity for distant metastasis, rapid progression, or even development of resistance to anti-androgen therapy, which subsequently leads to phenotypic plasticity and epithelial–mesenchymal transition to poorly differentiated neuroendocrine variants that are difficult to diagnose and treat [8,9,10]. In an effort to address this knowledge gap regarding PC, numerous diagnostic and therapeutic targets have been identified. Among these, gastrin-releasing peptide receptor (GRPR), also known as bombesin type 2 receptor, has emerged as a prominent target of interest. Gorica et al. demonstrated that GRPR (a 384-amino acid G protein-coupled receptor) is expressed in up to 63–100% of primary PC tumors, 86% of metastatic lymph node disease, and 53% of bone metastases [11].

During the development and progression of the disease, changes in the androgen receptor (AR) gene occur, including amplifications, mutations, and increased signaling, along with so-called splice variants, which play an essential role in the development of castration resistance [12]. Conditioning factors that increase AR splice variants involve the nuclear factor-kappa-beta (NFκβ) activation [12,13,14,15]. Interestingly, the GRPR activation has been shown to condition the activation pathway of NFκβ [15]. In addition, GRPR acts as a paracrine signaling pathway with mitogenic effects in PC, increasing the expression of metalloproteinases that remodel the extracellular matrix of the tumor microenvironment, allowing a greater capacity for tumor growth and invasion, resulting in mCRPC [12,13,14]. Thus, GRPR is a target with oncological diagnostic potential that has already been studied, both individually and as a dual target with PSMA (PET/CT), in the setting of biochemical recurrence and metastatic castration-resistant disease, with heterogeneous but encouraging results for further study of the duality of these molecules [14,16,17]. In addition, several attempts have already been made to use the GRPR as a target for therapeutic purposes, with modest but positive results [18,19,20].

Previously, our group reported the development of ^68^Ga- and ^177^Lu-labeled iPSMA-Lys^3^-Bombesin (iPSMA-BN) for theranostic dual targeting in PC [21,22]. [^68^Ga]-iPSMA-BN PET/CT was performed in humans, and adequate biokinetic, dosimetric, and lesion visualization parameters were observed [23].

Given the relatively high availability of SPECT/CT equipment worldwide, the objective of this study was to prepare and preclinically evaluate [^99m^Tc]Tc-iPSMA-BN, estimate dosimetry in healthy subjects, and assess the diagnostic efficacy of the radiotracer in patients with metastatic PC, with the hypothesis of non-inferiority to one of the gold standards, [^18^F]-PSMA-1007 [24]. In addition, the potential of [^99m^Tc]Tc-iPSMA-BN as a theranostic pair with [^177^Lu]Lu-iPSMA-BN was investigated.

## 2. Materials and Methods

### 2.1. Preparation and Preclinical Studies of iPSMA-Lys^3^-Bombesin (iPSMA-BN) Radioligands

#### 2.1.1. Synthesis and Radiolabeling of iPSMA-BN

The 6-hydrazinylnicotinoyl-iPSMA-BN (iPSMA-BN) was designed in our laboratory, where the Lys^3^-bombesin (1–14) peptide was attached through the primary amine of lysine to 4-(2,5-Dioxo-2,5-dihydro-1H-pyrrol-1-yl)butanoic acid (GMBS), which was used as a branch to form a thioether with the side chain of cysteine previously linked to iPSMA and HYNIC (6-hydrazinylnicotinamide): Glu-CO-Lys-Nal-Cys-HYNIC. For the scaled-up synthesis, we had the support of Ontores (Zhejiang, China). The complete structure was characterized with FT-infrared (ATR, PerkinElmer, Waltham, MA, USA) and mass spectroscopy (+Q1, turbo spray, LC/MS, Agilent, Santa Clara, CA, USA).

iPSMA-BN (50 µg) was prepared as a lyophilized formulation with ethylenediamine-*N*,*N′*-diacetic acid, stannous chloride, mannitol, and tricine in a GMP-certified facility (ININ, Ocoyoacac, Mexico) as previously reported by us [25]. For radiolabeling, the lyophilized formulation was reconstituted with 1.0 mL of 0.2 M phosphate buffer pH 7, followed by the addition of 1.0 mL (740 MBq) of [^99m^Tc]TcO_4_Na/0.9%NaCl eluted from a ^99^Mo/^99m^Tc generator (ININ, Ocoyoacac, Mexico) and finally incubated at 95 °C for 10 min. The radiochemical purity of [^99m^Tc]Tc-labeled HYNIC-iPSMA-BN ([^99m^Tc]Tc-iPSMA-BN) was determined via radio-HPLC (Shimadzu Corporation, Kyoto, Japan) using a C18 column (250 mm × 4.6 mm ID, Perkin-Elmer, Waltham, MA, USA) with a solvent system of 0.1% TFA/water and 0.1% TFA/acetonitrile in a linear gradient from 100 to 30% of the aqueous phase in 20 min at 1 mL/min. To evaluate the stability of [^99m^Tc]Tc-iPSMA-BN, it was diluted (5X) in mouse serum and incubated at 37 °C for 2 and 24 h, after which acetonitrile was added to precipitate proteins and the radiochemical purity of a sample of the supernatant was evaluated via radio-HPLC as described above.

For comparison purposes, [^99m^Tc]Tc-iPSMA, [^99m^Tc]Tc-BN, and [^177^Lu]Lu-iPSMA were prepared according to our previously reported methodology [25,26,27]. [^18^F]-PSMA-1007 for PET/CT imaging was obtained from the Cyclotron Unit (UNAM, Mexico City, Mexico).

#### 2.1.2. Preclinical Evaluation of [^99m^Tc]Tc-iPSMA-BN

- In vitro experiments

LNCaP and PC3 human prostate cancer cells (ATCC, Manassas, VA, USA) were used for preclinical evaluation because we have previously demonstrated via PCR assays that PSMA expression is 25-fold higher in LNCaP cells than in PC3 cells, while GRPR expression is 32-fold higher in PC3 cells than in LNCaP cells [22]. Both cell lines were cultured in RPMI medium (Mallinckrodt, St. Louis, MO, USA) with complements as described [22].

Cellular uptake of [^99m^Tc]Tc-iPSMA-BN was assessed in tubes (*n* = 6) containing 100,000 PC3 or LNCaP cells in a culture medium incubated with the radiopharmaceutical (185 kBq/12.5 ng per assay tube) at 37 °C for 1 h. The number of counts per minute (representing 100% of initial activity) in each tube was then determined using a NaI(Tl) detector (NML, St. Louis, MO, USA). Cells were centrifuged, washed with glycine solution (50 mM, pH 2.8) and centrifuged again. Tubes were decanted, and cell button counts per minute were correlated with initial activity to determine the percentage of radiopharmaceutical uptake in each cell line. For comparison, the same procedure was repeated for the radiopharmaceuticals [^99m^Tc]Tc-iPSMA and [^99m^Tc]Tc-BN. To verify the specificity of the cellular uptake, all experiments were repeated but with the addition of 10 µg of the non-radioactive peptides iPSMA-BN, iPSMA, and BN as a blocking procedure of cell-expressed receptors.

The in vitro affinity of the [^99m^Tc]Tc-iPSMA-BN heterodimer in both LNCaP and PC3 cells was determined with a competition assay. For this purpose, the cell lines were incubated in 96-well plates at 37 °C for 1 h with different concentrations of non-radioactive iPSMA-BN peptide (from 1000 to 0.06 nM; *n* = 3) at a constant concentration of [^99m^Tc]Tc-iPSMA-BN (18.5 kBq/1.25 ng). The plates were washed with a buffer solution pH 7.4 containing 120 mM NaCl, 25 mM Tris-HCl, 1 mM CaCl_2,_ and 0.1% BSA. Activity in each well was read on an NaI(Tl) detector. The uptake percentages in each well relative to the initial activity were fitted as competition curves to obtain the IC_50_ using GraphPad Prism Software (v. 10.3.1, 2024). For comparison, the IC_50_ of [^99m^Tc]Tc-bombesin in PC3 cells and the IC50 of [^99m^Tc]Tc-iPSMA in LNCaP cells were determined in parallel.

- In vivo experiments

In accordance with the ethical regulations of the National Standard for the Care of Laboratory (NOM-062-ZO-1999), the Institutional Committee for the Use and Care of Laboratory Animals (CICUAL-ININ) approved the animal experimental protocol (ID 11-2018-2022).

BALB/c nu mice (male athymic mice; 5 weeks old; 18–20 g weight; INCMNSZ, Mexico City, Mexico) were subcutaneously inoculated with one million PC3 cells on the left back and one million LNCaP cells on the right back to evaluate the in vivo biodistribution of [^99m^Tc]Tc-iPSMA-BN. When the tumors developed and became visible (0.4 to 0.45 cm^3^), 37 MBq of the radiopharmaceutical was administered via the caudal vein. After 1, 3, and 24 h (*n* = 3), the mice were sacrificed, and the organs and tissues of interest (lung, liver, pancreas, spleen, intestine, kidney, blood, and tumors) were dissected, weighed, and the activity evaluated in an NaI(Tl) detector to correlate the percentage of initially injected activity per gram of tissue (%ID/g). One group of animals was co-injected with [^99m^Tc]Tc iPSMA-BN and 100 µg non-radiolabeled iPSMA-BN (blocked receptor group). After 3 h, animals (*n* = 3) were sacrificed, and biodistribution was evaluated as described above. The biodistribution of [^99m^Tc]Tc-bombesin and [^99m^Tc]Tc-iPSMA was evaluated in parallel using the same procedure. One group of mice injected with [^99m^Tc]Tc-iPSMA-BN was anesthetized with 2% isoflurane. Images of tumor uptake (left dorsal LNCaP and right dorsal PC3) were obtained 2 h after radiopharmaceutical administration using a microPET/SPECT/CT (Albira, Bruker, Billerica, MA, USA).

- Studies on safety and biocompatibility of iPSMA-BN.

Safety testing was performed as described in the General Method of Analysis (MGA 0795) of the Mexican Pharmacopeia, 13th Edition. Healthy male mice of the BALB/c strain (*n* = 5), weighing 18 to 20 g, were used under sterile environmental conditions, controlled temperature, humidity, noise, 12:12 h light period, and standard PMI 5001 ad libitum feeding. Mice were weighed and administered 50 µL (50 µg) of iPSMA-BN (2.5 mg/kg) intraperitoneally, which is approximately 3500 times the human dose per kg body weight (0.0007 mg/kg). The animals were observed at 12 h intervals for 48 h, and their behavior and survival were evaluated. The mice were then sacrificed, and histological examination (H&E: hematoxylin-eosin staining) of kidneys, spleen, liver, lungs, and intestines was performed and compared with a control group (*n* = 5) of untreated mice. The blood samples obtained from the mice were used for creatinine quantification (titrimetric picrate method). Blood aspartate aminotransferase (AST), lactate dehydrogenase (LDH), and alanine aminotransferase (ALT) were quantified via colorimetric assays (Diagnostic Kits; Merck, Saint Louis, MO, USA) and compared with those of the control group.

### 2.2. Clinical Evaluation

#### 2.2.1. Biokinetics and Dosimetry

To evaluate the effective radiation dose and biokinetics of [^99m^Tc]Tc-iPSMA-BN, six healthy volunteers were recruited: three males (ages 29, 34, and 36 years) and three females (ages 30, 33, and 37 years) with no known medical conditions. They consented to this study (prior written informed consent), which consisted of four phases: a whole-body (WB) planar image 30 min after radiopharmaceutical injection (740 MBq), with no voiding until the first image was acquired; WB images at 1 h, 5 h, and 24 h; and a three-dimensional hybrid SPECT/CT image (Symbia TP; Siemens, Munich, Germany) along with low-dose CT for attenuation correction in the fourth phase of this study. Images were acquired with 360° rotation, a 128 × 128 matrix, with the photopeak window centered at 140 keV scatter correction, and sixty images of ten seconds each. Volumes of interest (VOIs) were drawn around the area of each source organ. Hybrid 2D-planar/3D-SPECT activity quantification was used to fit biokinetic models as previously reported [28]. Correction factors (CFs) between 2D (planar images acquired at 0.5 h, 1 h, 5 h, and 24 h) and 3D (SPECT/CT was acquired at 24 h) images were then calculated by dividing the activity in the source tissues quantified using 3D-SPECT (reconstructions in kBq/cm^3^) via the activity measured in the 2D planar images. The CFs obtained for the source organs were used in the quantification of the A(t)P (planar process) biokinetic models for the construction of the A(t)VOI (volumetric activity) 3D biokinetic models, which, after mathematical integration (from t = 0 to t = ∞), give the total number of nuclear transitions (N) in the total volume of each source organ. The N values were used to estimate the dosimetry of [^99m^Tc]Tc-iPSMA-BN using the OLINDA/EXM code. The [^99m^Tc]Tc-iPSMA-BN biokinetic models were corrected using the lutetium-177 decay constant to obtain an estimated [^177^Lu]Lu-iPSMA-BN dosimetry.

#### 2.2.2. Imaging in Patients

In this study, [^99m^Tc]Tc-iPSMA-BN SPECT/CT clinical evaluation was performed and compared with an FDA-approved standard for PC molecular imaging (^18^F-PSMA-1007 PET/CT) in 11 patients with metastatic PC to detect primary lesion, locoregional metastases, and distant metastases; 36.36% (*n* = 4/11) of them were hormone sensitive with de novo metastatic disease versus 63.63% (*n* = 7/11) of men who had a long known diagnosis with castration-resistant hormonal status. All patients signed a written informed consent form, and all of them were classified as high and very high risk according to the National Comprehensive Cancer Network (NCCN) guideline for diagnosing and managing this type of neoplasia [29]. Patients without a histologically confirmed diagnosis of prostate adenocarcinoma or non-acinar cell-derived subtypes and those with functional status 3 on the ECOG scale were excluded. Patients with hemoglobin < 8.0 g/dL, leukocytes < 2000/mm^3^, platelets < 75,000/mm^3^, total bilirubin > 3 times the upper limit of normal, serum albumin > 3.0 g/dL, serum creatinine > 1.7 mg/dL, and life expectancy < 6 months were also excluded. Withdrawal of informed consent was also an exclusion criterion. The clinical and pathologic characteristics of patients are presented in Table 1. All patients received 740 MBq [^99m^Tc]Tc-iPSMA-BN intravenously. The protocol established for the [^99m^Tc]Tc-iPSMA-BN clinical trial adhered to the ethical codes of professional conduct, objectivity, integrity, competence, and confidentiality. Patients were informed that their participation was voluntary and that their identity and medical information would be kept confidential.

The hospital’s approval of the protocol was based on the Helsinki Declaration of 1975 (revised in 2008); the GMP certificate issued by COFEPRIS (Federal Commission for the Protection against Health Risks, regulatory authority in Mexico) to the ININ facilities where the radiopharmaceuticals were prepared; and the prior approval by COFEPRIS for human use of [^99m^Tc]Tc-bombesin (registration number 0004R2009 SSA), [^99m^Tc]Tc-iPSMA (registration number 2764R2017 SSA), and [^177^Lu]Lu-iPSMA (registration number 2763R2017 SSA).

Patients underwent [^18^F]-PSMA-1007 imaging with a PET/CT scanner (Siemens E20, Munich, Germany) at a maximum interval of 30 days (average 18 days) before the [^99m^Tc]Tc-iPSMA-BN SPECT/CT study.

[^99m^Tc]Tc-iPSMA-BN and ^18^F-PSMA-1007 images were semi-quantitatively analyzed by two nuclear medicine physicians using volumetric analysis software (Siemens VG60 multimodality workstation). Tumor-to-background ratios (TBRs) were calculated by setting volumetric regions of interest (VOI) around tumor (T) and healthy tissue as background (B). Activity in VOIs was expressed as Bq/cm^3^ for [^99m^Tc]Tc-iPSMA-BN images and as maximum standardized uptake value (SUVmax) for [^18^F]-PSMA-1007 images.

## 3. Results

### 3.1. Preparation and Preclinical Studies of iPSMA-BN Radioligands

#### 3.1.1. Chemical and Radiochemical Results

**Figure 1:**
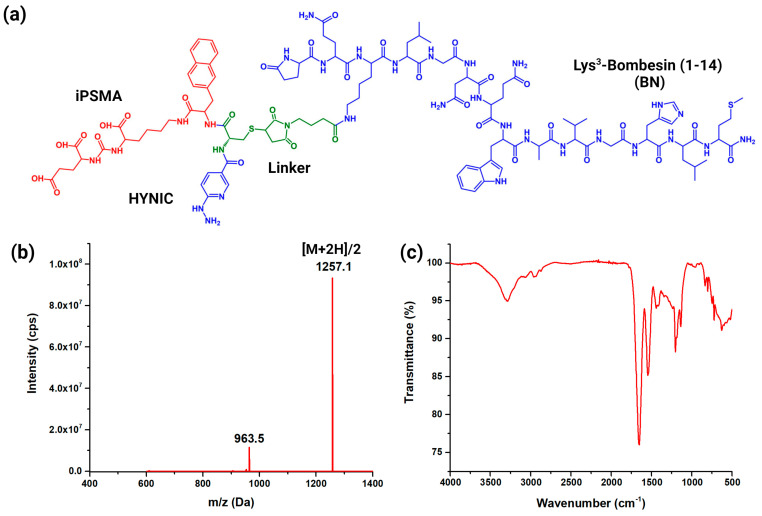
(**a**) Schematic chemical structure of iPSMA-BN with HYNIC for ^99m^Tc labeling, (**b**) mass, and (**c**) FT-IR spectra of iPSMA-BN.

Chemical characterization of iPSMA-BN (Figure 1a) was performed via mass spectroscopy (Figure 1b), which revealed the molecular ion [M + 2H]/2 at *m*/*z* 1257.1 Da. The fragment from pyridine-GMBS-BN was also identified as [M + 2H]/2 at 963.5 Da. FT-IR spectroscopy (Figure 1c) confirmed the presence of the characteristic bands of amide I (1650 cm^−1^: C=O stretching vibration) and amide II (1544 cm^−1^: N-H bending and C-N stretching vibrations) normally found in peptide molecules. The amide IV band related to the -CO-NH- moiety was observed at 628 cm^−1^. The antisymmetric stretching of -NH_2_ was observed at 3290 cm^−1^. In addition, the vibrational band at 1201 cm^−1^ from the amide II region of bombesin was assigned to N-H bending and C-N stretching vibrations [30]. The C-N stretching vibration of urea from iPSMA was observed at 1440 cm^−1^. The stretching vibration of the N-N bond corresponded to hydrazinopyridine at 1137 cm^−1^ [25].

Radio-HPLC results showed that [^99m^Tc]Tc-iPSMA-BN (Tr = 13.8 min) was obtained with a radiochemical purity (RP) of 97 ± 1% (*n* = 15), which remained practically unchanged in blood serum medium after 2 h (RP = 96 ± 2%) and 24 h (PR 93 ± 2%) of its preparation.

#### 3.1.2. Preclinical Evaluation of [^99m^Tc]Tc-iPSMA-BN

**Figure 2:**
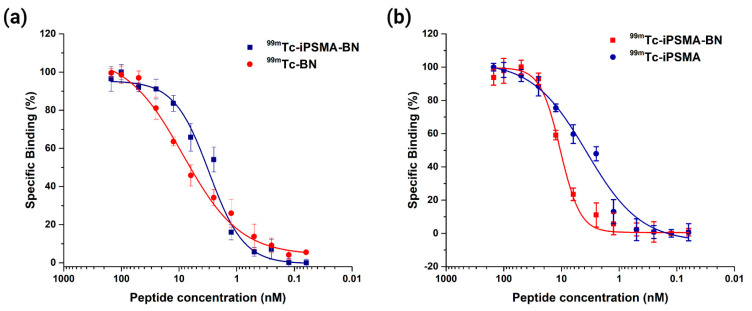
Competition binding assay in (**a**) PC3 cells of [^99m^Tc]Tc-iPSMA-BN and [^99m^Tc]Tc–BN and (**b**) LNCaP cells of [^99m^Tc]Tc-iPSMA-BN and [^99m^Tc]Tc-iPSMA.

The cell binding assay revealed an IC50 value for the [^99m^Tc]Tc-iPSMA-BN heterodimer of 3.24 nM (95% CI 2.51 nM–4.39 nM) in PC3 cells (Figure 2a) and 10.45 nM (95% CI 9.59 nM–11.29 nM) in the LNCaP cell line (Figure 2b), indicating adequate affinity for GRPR and PSMA receptors. Compared to the monomer, [^99m^Tc]Tc-iPSMA showed higher affinity than the heterodimer with an IC50 in LNCaP cells of 3.83 nM (95% CI 2.89 nM–4.79 nM), while [^99m^Tc]Tc-BN showed lower affinity than [^99m^Tc]Tc-iPSMA-BN in the PC3 cell line with an IC50 of 6.39 nM (95% CI 5.10 nM–7.71 nM) (Figure 2).

When comparing IC50 values with other bispecific peptides targeting PSMA/GRPR, it is noted that for both receptors, [^99m^Tc]Tc-iPSMA-BN showed values < 10.5. In contrast, for the Glu-urea-Lys-HBED-CC-BZH3 dimer, IC50 values of 250 ± 54 nM for PSMA and 90 ± 18 nM for GRPR were demonstrated [31]. The dimeric peptide NOTA-DUPA-RM26 showed an IC50 of 4 ± 1 nM against GRPR and 824 ± 230 nM against PSMA [9]. Based on these evaluations, the iPSMA-BN heterodimeric peptide demonstrated enhanced protein–protein interactions in both cell lines with high specificity and selectivity in the detection of PSMA and GRP receptors.

**Figure 3:**
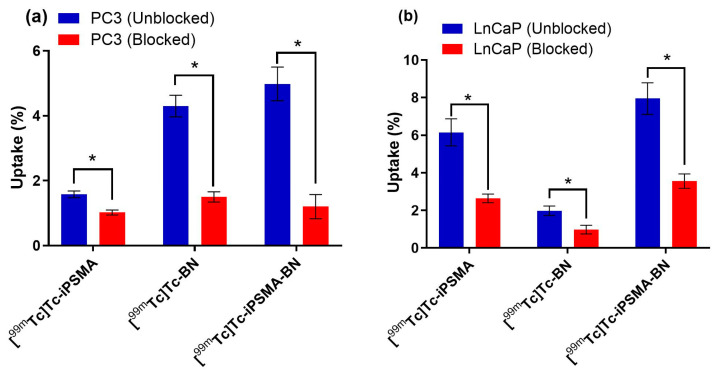
Cellular uptake of [^99m^Tc]Tc-iPSMA, [^99m^Tc]Tc-BN, and [^99m^Tc]Tc-iPSMA-BN in unblocked and blocked (**a**) PC3 and (**b**) LNCaP prostate cancer cells. Blocking:10 µg unlabeled iPSMA, BN, or iPSMA-BN peptides. * Statistically significant difference (*p* < 0.05, Mann–Whitney test).

The uptake of the [^99m^Tc]Tc-iPSMA-BN heterodimer in both PC3 and LNCaP cells was found to be higher than its [^99m^Tc]Tc-BN and [^99m^Tc]Tc-iPSMA monomers, with a significant decrease in uptake (*p* < 0.05, Mann–Whitney test) when the PSMA and GRPR receptors of the cells were blocked with an excess of non-radioactive peptide, indicating that the uptake of the radiopharmaceuticals is specific (Figure 3). At this point, it is important to reiterate that according to our previous PCR studies, PC3 cells express 25-fold less PSMA than LNCaP cells, whereas GRPR is expressed 32-fold more in PC3 cells than in LNCaP cells [22].

**Figure 4:**
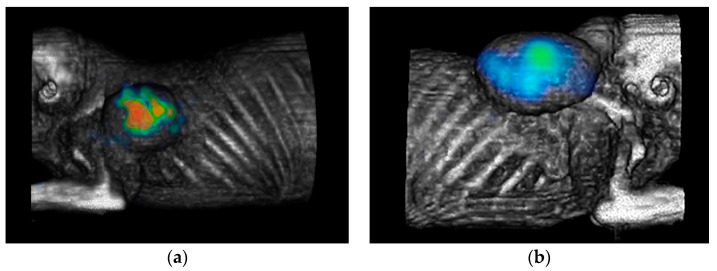
Micro-SPECT image of a mouse with induced (**a**) PC3 (right rear) and (**b**) LNCaP (left rear) tumors showing that [^99m^Tc]Tc-iPSMA-BN is taken up by both PSMA-expressing (LNCaP) and GRPR-expressing (PC3) tumors. Radiation intensity is indicated by the rainbow scale.

Ex vivo biodistribution studies and micro-SPECT imaging in mice with induced PC3 (right back) and LNCaP (left back) tumors showed that [^99m^Tc]Tc-iPSMA-BN is taken up in both PSMA-expressing and GRPR-expressing tumors (Table 2) (Figure 4).

The [^99m^Tc]Tc-iPSMA-BN tumor uptake is specific as it was significantly decreased (*p* < 0.05, t-student) in mouse tumors given a blocking dose via administration of 100 µg of non-radiolabeled iPSMA-BN peptide (Table 3). Compared to the monomers, the [^99m^Tc]Tc-iPSMA-BN shows a lower hepatic uptake than [^99m^Tc]Tc-iPSMA and a renal elimination profile like [^99m^Tc]Tc-BN. Therefore, the lutetium-177-labeled iPSMA-BN heterodimer would be expected to deliver an intermediate radiation-absorbed dose in the liver and kidneys between those obtained with ^177^Lu-iPSMA and ^177^Lu-BN.

A noteworthy point in the biodistribution is the evident uptake in the pancreas (high GRPR expression) of both [^99m^Tc]Tc-BN and [^99m^Tc]Tc-iPSMA-BN (Table 2), which significantly decreased in blocked mice (Table 3), again demonstrating the specific recognition of both radiopharmaceuticals by GRP receptors.

Safety test results showed that at the end of the observation period (48 h), the weight of each mouse did not change. The safety study was considered satisfactory since 100% of the animals survived, and no signs of toxicity were observed. Histological analysis (H&E staining) of organs from iPSMA-BN ligand-treated mice showed no histopathological abnormalities. There was no evidence of liver enzyme damage, as ALT (82 ± 9 IU/L), AST (151 ± 7 IU/L), and LDH (295 ± 31 IU/L) levels were not significantly different from those in the control group (76 ± 12 IU/L, 148 ± 10 IU/L, and 301 ± 25 IU/L, respectively). Similarly, renal function was not affected (creatinine levels in treated mice of 0.189 ± 0.057 mg/dL vs. 191 ± 0.064 mg/dL in control mice).

### 3.2. Clinical Evaluation of iPSMA-BN Radioligands

#### 3.2.1. Biokinetics and Dosimetry

**Figure 5:**
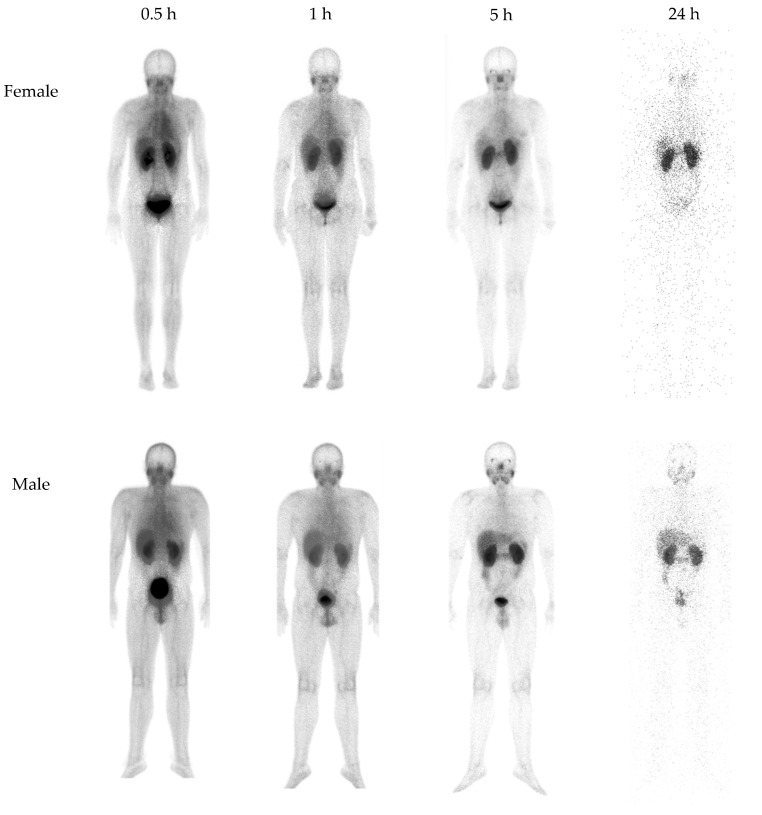
Time course of planar [^99m^Tc]Tc-iPSMA-BN anterior images of healthy subjects (740 MBq). As a proof of the recognition of [^99m^Tc]Tc-iPSMA-BN by PSMA and GRP receptors, both salivary gland (PSMA) and pancreas (GRPR) were visualized.

Some of the side effects reported by healthy volunteer subjects within the first 10 min after injection of [^99m^Tc]Tc-iPSMA-BN were the following: metallic taste sensation, sensation of heat in the injected arm, and nausea in those who had ingested food prior to the study, while those who remained fasting until after the radiopharmaceutical administration did not present this symptom; all these were grade 1 adverse effects, according to the Common Terminology Criteria for Adverse Events (CTCAE) [32], and had a duration of seconds to a couple of minutes at most in all cases. As proof of the recognition of [^99m^Tc]Tc-iPSMA-BN by PSMA and GRP receptors, both the salivary gland (PSMA) and pancreas (GRPR) were visualized, although only in late images (5 h) due to the high background activity coming mainly from the kidneys at early images (Figure 5).

The biokinetic models of source organs obtained from images are shown in Table 4. Thus, the estimated radiation doses shown in Table 5 indicate that the radiopharmaceutical [^99m^Tc]Tc-iPSMA-BN is radiologically safe because it delivers an effective radiation dose for the activity required in a diagnostic study (740 MBq) of less than 10 mSv [33]. In fact, the radiation dose of 1.94–2.37 mSv is equivalent to the natural background radiation dose received in one to two years (low radiation doses) [34].

#### 3.2.2. Imaging in Patients

**Figure 6:**
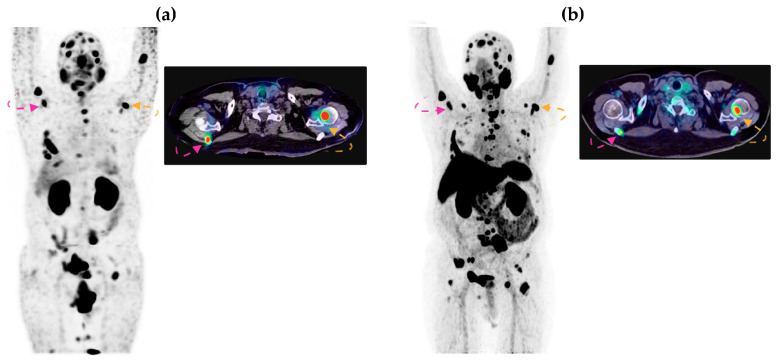
Patient 2: (**a**) [^99m^Tc]Tc-iPSMA-BN MIP (**left**) and SPECT/CT axial fusion (**right**) images; (**b**) [^18^F]-PSMA-1007 MIP (**left**) and PET/CT axial fusion (**right**) images. A higher concentration was observed in the axial fusion SPECT/CT projection compared to PET/CT. MIP: Maximum Intensity Projection. Radiation intensity is indicated by the rainbow scale. Yellow and red arrows indicate axillar lymph node lesions.

**Figure 7:**
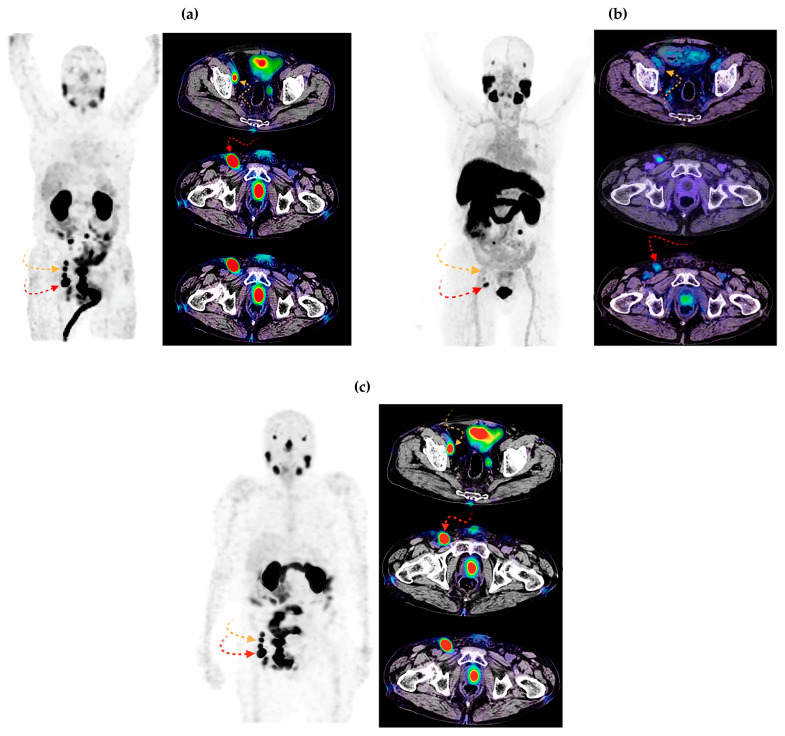
Patient 5: (**a**) [^99m^Tc]Tc-iPSMA-BN MIP (**left**) and SPECT/CT axial fusion (**right**) images; (**b**) [^18^F]-PSMA-1007 MIP (**left**) and PET/CT axial section (**right**) images; (**c**) [^177^Lu]Lu-iPSMA-BN MIP (**left**) and SPECT/CT axial section (**right**) images. [^99m^Tc]Tc-iPSMA-BN and [^177^Lu]Lu-iPSMA-BN showed lymphadenopathy with a high focal concentration in the right obturator chain, whereas [^18^F]-PSMA-1007 showed no concentration. MIP: Maximum Intensity Projection. Radiation intensity is indicated by the rainbow scale. Yellow and red arrows indicate lymph node lesions.

A total of 178 lesions were detected via CT, 162 via ^18^F-PSMA-1007 PET, and 155 via [^99m^Tc]Tc-iPSMA-BN SPECT (Table 6). Three patients (patients 2, 5, and 8) had higher TBR values and more locoregional lymph node lesions (patients 5 and 8) visualized via SPECT heterodimer than via PSMA-PET (Figure 6). The histopathological report of patient 5 was a dedifferentiated sarcomatoid variant and acinar adenocarcinoma ISUP grade 5 since diagnosis. NKX3.1 immunohistochemistry was performed and reported as positive; in addition, PSA at the time of study in this patient was 4.0 ng/dL, supporting loss of cellular differentiation and further PSA production; interestingly, this was the patient in whom we found a better lesion detection rate and higher TBR in the SPECT vs. PET study (86.66% vs. 73.33% and 207 vs. 25.31, respectively) (Figure 7).

**Figure 8:**
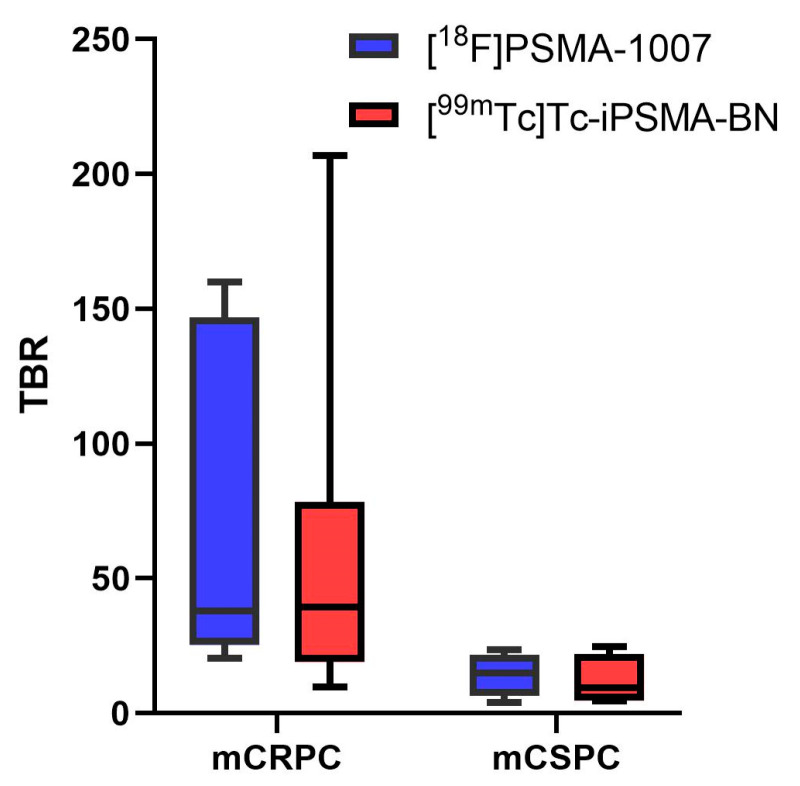
TBR values of prostatic lesions for [^99m^Tc]Tc–iPSMA–BN SPECT vs. [^18^F]–PSMA-1007 PET in patients with metastatic castration-resistance prostate cancer (mCRPC) and metastatic castration-sensitive prostate cancer (mCSPC). Two-way ANOVA statistical analysis showed a significant difference in TBR values between patients with mCRPC and mCSPC (*p* < 0.05) but no significant difference between the radiotracers [^18^F]-PSMA-1007 and [^99m^Tc]Tc-iPSMA-BN (*p* > 0.05).

In general, the clinical and imaging interpretation of the results obtained with [^99m^Tc]Tc-iPSMA-BN fit the more aggressive spectrum of long-standing disease, as the heterodimer had similar or, in some cases, inferior performance to [^18^F]-PSMA-1007 in patients with newly diagnosed neoplasia and clinically staged IV, with de novo metastases. However, in patients with metastatic castration-resistant hormone status, the heterodimer proved to be superior even in cases where aggressive histologic transformation has occurred, and PSA is no longer an appropriate marker representing tumor burden (e.g., patient 5). As expected, the two-way ANOVA statistical analysis revealed a significant difference in TBR values between patients with mCRPC and mCSPC (*p* = 0.0370) but no significant difference between the radiopharmaceuticals [^18^F]-PSMA-1007 and [^99m^Tc]Tc-iPSMA-BN (*p* = 0.8448) (Figure 8).

The biokinetic models of [^99m^Tc]Tc-iPSMA-BN obtained from healthy male volunteers were corrected using the lutetium-177 decay constant to obtain estimated dosimetry data of [^177^Lu]Lu-iPSMA-BN to evaluate the possibility of administering therapeutic treatment to patient 5. As shown in Table 7, the liver (0.070 mGy/MBq) and spleen (0.065 mGy/MBq) absorbed doses were 4- and 3.6-times lower, respectively, than the previously reported values for [^177^Lu]Lu -iPSMA (0.280 mGy/MBq for liver and 0.232 for spleen) [35]; while the renal dose remained in the same order of magnitude between the heterodimer and the monomer radiopharmaceuticals (0.97 mGy/MBq vs. 0.88 mGy/MBq), the dose in the salivary and lacrimal glands was reduced by approximately 20 times [35]. Indeed, patient 5 received the [^177^Lu]Lu -iPSMA-BN dose (Figure 7c), which showed an excellent correlation with the [^99m^Tc]Tc-iPSMA-BN theranostic pair (Figure 7a). However, the treatment was not continued as the patient was diagnosed with Fournier’s gangrene.

## 4. Discussion

The preclinical and clinical studies presented in this research demonstrated that the iPSMA-BN radioligands, [^99m^Tc]Tc-iPSMA-BN and [^177^Lu]Lu-iPSMA-BN, have the ability to detect in vivo expression of both PSMA and GRP receptors for potential theranostic application in patients with metastatic PC. As previously reported, the specific recognition of the heterodimeric molecule is based on a proper orientation, without steric hindrance, of the two active sites responsible for the recognition, as a result of an optimal improper torsional energy, which typically controls the affinity by strengthening the planar bonds [22].

Lutetium-177-bombesin analogs have been proposed as a therapeutic alternative for patients with low and/or heterogeneous PSMA expression [18,20]. This assertion is supported by the results obtained from a comparative study between [^68^Ga]-PSMA-11 and [^68^Ga]-RM2 (GRPR detection) in patients with mCRPC, which have shown that [^68^Ga]-PSMA-11 tumor uptake was superior to that of [^68^Ga]-RM2 in most mCRPC lesions [16]. Therefore, GRPR expression has traditionally been associated with low-grade disease, as various GRP agonist and antagonist radioligands have shown promising results in the detection of early-stage prostate cancer [36]. However, consistent with this research, Verhoeven et al. demonstrated the added value of GRPR-targeted radioligands in mCRPC theranostics by showing uptake of [^177^Lu]Lu-NeoB (GRP radioligand) in CRPC tissue where [^177^Lu]Lu-PSMA-617 did not bind [37]. GRPR activation influences androgen receptors and its splice variants through nuclear factor-kappa-beta, involved in developing castration resistance and progression of PC [15]. Therefore, it is evident that GRPR expression can be found in aggressive mCRPC in which homologous recombination repair germline and somatic mutations may occur.

In this research, the novel heterodimer [^99m^Tc]Tc-iPSMA-BN was shown to outperform [^18^F]-PSMA-1007 in the more aggressive spectrum of long-standing disease, cases in which tumors no longer produce PSA, and, therefore, it is no longer an appropriate tumor marker to represent tumor burden. In patients with newly diagnosed neoplasia and staged de novo metastasis, the heterodimer had similar or, in some cases, inferior performance to [^18^F]-PSMA-1007, although without a statistically significant difference in TBR (*p* > 0.05).

The [^18^F]-PSMA-1007 radiotracer is currently considered one of the gold standards for staging distant disease in patients with unfavorable intermediate- and high-risk prostate cancer. This pilot study comparing [^18^F]-PSMA-1007 PET/CT with [^99m^Tc]Tc-iPSMA-BN SPECT/CT in 11 patients diagnosed with metastatic prostate cancer showed no significant difference (*p* < 0.05) in the target-to-background ratio of volumetric lesions between the two imaging modalities. This shows that although SPECT devices have lower sensitivity and spatial resolution compared to PET, the utility of SPECT/CT is validated by the specificity of the developed radioligands, such as the [^99m^Tc]Tc-iPSMA-BN heterodimer.

Given that GRPR-targeted radiotracers are useful in the diagnosis of low-grade PC disease and in aggressive mCRPC (PSMA absent), and that PSMA-targeted radiotracers are highly efficient in the detection of mCRPC lesions, then, consistent with the results of this study, the iPSMA-BN heterodimer is achieving the goal for which it was designed by broadening the spectrum of PC patients for whom radionuclide theranostics may be beneficial. Clearly, this pilot study’s results are preliminary, as the main limitation is the small number of patients included. Nevertheless, the results of this investigation justify the development of a clinical trial involving a significant number of patients diagnosed with metastatic prostate cancer.

## 5. Conclusions

The [^99m^Tc]Tc-iPSMA-BN heterodimer proved to be a safe radiotracer with the ability to detect both PSMA and GRP receptors in patients with metastatic prostate cancer. Cancer lesion uptake values (TBR) obtained with [^99m^Tc]Tc-iPSMA-BN SPECT/CT were not statistically inferior to those obtained with [^18^F]-PSMA-1007 PET/CT. [^99m^Tc]Tc-iPSMA-BN appears to have better lesion detection in patients with aggressive histologic transformation. The high correlation found between [^99m^Tc]Tc-iPSMA-BN and [^177^Lu]Lu-iPSMA-BN of radioactivity concentrated in the lesions of a prostate cancer patient is indicative of the theranostic potential of the iPSMA-BN radioligands. [^99m^Tc]Tc-iPSMA-BN SPECT/CT is a promising alternative not only for diagnostic purposes with wider availability, but also for more patients to undergo radionuclide therapy and not be excluded from this treatment modality.

## 6. Patents

Radiolabeled (Lys^3^)BN-iPSMA for the dual recognition of the PSMA and GRPR proteins in vivo. Mexican Patent ZA202205596 and MX2019012648.

## Figures and Tables

**Table 1:** Patient characteristics before PET/CT and SPECT/CT molecular imaging studies.

Patient No.	Age (Years)	Gleason Score	Histology	PSA (ng/mL) at Diagnosis [at the Time of Study]	Status of the Disease [Time Since Diagnosis]	Genetic Alterations	Previous Treatments
1	58	9 (4 + 5)	Acinar adenocarcinoma.	108 [14.1]	mCRPC [2 y]	Positive germline HRR PV mutation in *ATM* gene	Radiotherapy (primary), Gosereline + Enzalutamide, Docetaxel
2	75	8 (4 + 4)	Acinar adenocarcinoma.	40.9 [440.6]	mCRPC [7 y]	No known PV in germline HRR genes, germline VUS in *CHEK2*	Leuproreline, Enzalutamide, Abiraterone + predinsone, Docetaxel, Cabazitaxel
3	62	9 (4 + 5)	Acinar adenocarcinoma.	39.8 [96.7]	mCRPC [2 y]	No known PV in germline HRR genes, germline VUS in *BRCA*	Bicalutamide, Gosereline, Enzalutamide, Docetaxel, Cabazitaxel
4	77	9 (4 + 5)	Acinar adenocarcinoma.	6.0 [11.7]	mCRPC [17 m]	Positive germline HRR PV mutation + somatic mutation in *p53*	Bilateral orchiectomy, abiraterone + prednisone, Docetaxel, Cabazitaxel
5	77	8 (4 + 4)	Acinar adenocarcinoma. Sarcomatoid differentiation on 2° biopsy 10 years later.	34.5 [4.0]	mCRPC [11 y]	Positive germline HRR PV mutation in *ATM* gene	Radiotherapy (primary), Cyproterone, Gosereline, Enzalutamide, Docetaxel
6	73	9 (4 + 5)	Acinar adenocarcinoma.	491.2 [11.1]	mCSPC (de novo) [4 m]	Negative germline HRR mutations	Bicalutamide, Gosereline, Apalutamide
7	78	N/A	Metastatic acinar adenocarcinoma on bladder and rectum biopsies.	266.5 [8.9]	mCRPC [2 y]	Negative germline HRR mutations	Radiotherapy (primary), Degarelix, Enzalutamide, Docetaxel
8	72	9 (4 + 5)	Acinar adenocarcinoma.	96 [100.5]	mCRPC [2 m]	Negative germline HRR mutation	Bilateral orchiectomy, Docetaxel
9	64	9 (4 + 5)	Acinar adenocarcinoma.	9.32 [1.3]	mCSPC (de novo, oligo) [4 m]	Negative germline HRR mutation	Gosereline + Enzalutamide
10	63	9 (4 + 5)	Acinar adenocarcinoma.	875.2 [49.0]	mCSPC (de novo) [3 m]	Negative germline HRR mutation	Holep Enucleation, Gosereline, Abiraterone + prednisone,
11	71	N/A	Acinar adenocarcinoma.	222 [0.16]	mCSPC (de novo) [5 m]	Negative germline HRR mutation	Bilateral orchiectomy, bicalutamide, carboplatin/paclitaxel

mCRPC: metastatic castration-resistant prostate cancer; mCSPC: metastatic castration-sensitive prostate cancer; HRR: homologous recombination repair; PV: pathogenic variant; VUS: variant of uncertain significance; y: year; m: month.; N/A: not available.

**Table 2:** Time course biodistribution (%ID/g) of [^99m^Tc]Tc-iPSMA, [^99m^Tc]Tc-BN, and [^99m^Tc]Tc-iPSMA-BN in athymic mice bearing PC3 and LNCaP tumors.

Organ/Tissue	Time (h)	[^99m^Tc]Tc-iPSMA	[^99m^Tc]Tc-BN	[^99m^Tc]Tc-iPSMA-BN
Blood	1	1.08 ± 0.12	1.14 ± 0.09	1.02 ± 0.15
	3	0.82 ± 0.10	0.75 ± 0.11	0.74 ± 0.07
	24	0.25 ± 0.01	0.18 ± 0.01	0.14 ± 0.01
Liver	1	3.44 ± 0.48	0.75 ± 0.21	1.42 ± 0.14
	3	2.29 ± 0.35	0.61 ± 0.12	1.08 ± 0.12
	24	1.75 ± 0.24	0.11 ± 0.01	0.28 ± 0.07
Kidney	1	15.95 ± 1.67	18.48 ± 1.92	20.41 ± 2.12
	3	10.23 ± 1.42	13.56 ± 1.98	14.57 ± 1.81
	24	3.87 ± 0.69	7.46 ± 1.02	5.12 ± 0.87
Spleen	1	1.78 ± 0.41	0.54 ± 0.23	0.86 ± 0.34
	3	1.29 ± 0.28	0.28 ± 0.11	0.32 ± 0.13
	24	0.95 ± 0.18	0.11 ± 0.02	0.14 ± 0.01
Lung	1	0.64 ± 0.12	0.51 ± 0.18	0.58 ± 0.11
	3	0.29 ± 0.08	0.22 ± 0.05	0.18 ± 0.09
	24	0.13 ± 0.07	0.11 ± 0.02	0.12 ± 0.01
Intestine	1	0.63 ± 0.14	0.45 ± 0.17	0.51 ± 0.12
	3	0.84 ± 0.13	0.31 ± 0.10	0.49 ± 0.17
	24	0.75 ± 0.18	0.15 ± 0.04	0.45 ± 0.08
Pancreas	1	0.21 ± 0.12	3.41 ± 0.51	3.21 ± 0.28
	3	0.11 ± 0.02	2.98 ± 0.32	3.02 ± 0.44
	24	0.08 ± 0.01	1.85 ± 0.36	1.75 ± 0.30
PC3 Tumor (Unblocking)	1	1.44 ± 0.54	6.23 ± 1.27	5.25 ± 0.98
	3	0.85 ± 0.18	5.72 ± 0.85	4.61 ± 0.79
	24	0.35 ± 0.07	3.38 ± 0.69	3.13 ± 0.71
LNCaP Tumor (Unblocking)	1	9.32 ± 1.32	1.11 ± 0.16	10.14 ± 1.25
	3	8.87 ± 1.01	0.88 ± 0.11	9.33 ± 1.14
	24	4.95 ± 0.85	0.41 ± 0.07	5.41 ± 1.02

**Table 3:** Biodistribution (%ID/g) at 3 h of [^99m^Tc]Tc-iPSMA, [^99m^Tc]Tc-BN, and [^99m^Tc]Tc-iPSMA-BN in athymic mice bearing PC3 and LNCaP tumors and administered with a blocking dose of 100 µg of non-radiolabeled iPSMA-BN peptide.

Organ/tissue	[^99m^Tc]Tc-iPSMA	[^99m^Tc]Tc-BN	[^99m^Tc]Tc-PSMA-BN
Blood	0.91 ± 0.24	0.68 ± 0.09	0.82 ± 0.13
Liver	2.63 ± 0.29	0.74 ± 0.11	1.13 ± 0.18
Kidney	11.47 ± 1.61	15.25 ± 1.75	14.98 ± 1.69
Spleen	1.14 ± 0.33	0.34 ± 0.14	0.21 ± 0.14
Lung	0.21 ± 0.12	0.17 ± 0.08	0.24 ± 0.11
Intestine	1.01 ± 0.17	0.52 ± 0.15	0.51 ± 0.21
Pancreas	0.13 ± 0.01	1.23 ± 0.28 *	0.88 ± 0.12 *
PC3 Tumor (Blocking)	0.57 ± 0.10 *	2.08 ± 0.44 *	1.58 ± 0.36 *
LNCaP Tumor (Blocking)	2.28 ± 0.52 *	0.61 ± 0.28 *	3.62 ± 0.63 *

* Statistically significant difference in %ID/g at 3 h post-injection compared to the organ or tumor in the unblocked mice showed in Table 2 (*p* < 0.05, t-student).

**Table 4:** Biokinetic models (mean) of [^99m^Tc]Tc-iPSMA-BN and total nuclear transformations (N) (MBq^.^h/MBq) in source organs (mean ± SD) estimated from six healthy volunteers (three female and three male) using a hybrid imaging methodology (2D/SPECT).

Gender	Organ	Biokinetic Model ${A(t)}_{VOI}$	$N=\int_{t=0}^{t=\infty }{A(t)}_{VOI}dt$
Female	Liver	${A(t)}_{VOI}={13.20e}^{-2.34t}+{5.42e}^{-0.18t}$ R^2^ = 1	3.35 × 10^−1^ ± 8.25 × 10^−2^
	Kidneys	${A(t)}_{VOI}={17.30e}^{-1.52t}+{9.74e}^{-0.14t}$ R^2^ = 1	7.55 × 10^−1^ ± 1.27 × 10^−1^
	Urinary Bladder	${A(t)}_{VOI}=11.60e^{-0.96t}+2.78e^{-0.27t}$ R^2^ = 1	2.25 × 10^−1^ ± 1.37 × 10^−2^
	Pancreas	${A\left(t\right)}_{VOI}=0.71\;e^{-0.74t}+0.60e^{-0.17t}$ R^2^ = 1	3.85 × 10^−2^ ± 1.90 × 10^−2^
	Lacrimal glands	${A(t)}_{VOI}=1.02\;e^{-6.38t}+0.16e^{-0.46t}$ R^2^ = 0.99	4.37 × 10^−3^ ± 2.18 × 10^−3^
	Salivary glands	${A(t)}_{VOI}=1.32\;e^{-1.03t}+0.62e^{-0.19t}$ R^2^ = 1	4.59 × 10^−2^ ± 8.46 × 10^−3^
	Remainder of the body	${A\left(t\right)}_{VOI}=58.60^{-0.94t}+{21.30e}^{-0.19t}$ R^2^ = 1	1.58 ± 11.91 × 10^−1^
Male	Liver	${A(t)}_{VOI}={7.20e}^{-0.59t}+{3.78e}^{-0.17t}$ R^2^ = 1	3.36 × 10^−1^ ± 1.11 × 10^−2^
	Kidneys	${A(t)}_{VOI}={9.65e}^{-0.98t}+{9.54e}^{-0.16t}$ R^2^ = 1	6.76 × 10^−1^ ± 5.96 × 10^−2^
	Urinary Bladder	${A(t)}_{VOI}=5.01e^{-2.14t}+5.75e^{-0.26t}$ R^2^ = 1	2.32 × 10^−1^ ± 1.22 × 10^−1^
	Pancreas	${A\left(t\right)}_{VOI}=1.91e^{-2.19t}+1.10e^{-0.20t}$ R^2^ = 0.99	6.38 × 10^−2^ ± 3.03 × 10^−2^
	Lacrimal glands	${A(t)}_{VOI}=0.22\;e^{-42.92t}+0.07e^{-0.34t}$ R^2^ = 0.98	2.73 × 10^−3^ ± 1.10 × 10^−3^
	Salivary glands	${A(t)}_{VOI}=0.63e^{-5.15t}+1.02e^{-0.37t}$ R^2^ = 0.99	2.93 × 10^−2^ ± 8.20 × 10^−3^
	Remainder of the body	${A\left(t\right)}_{VOI}={49.30e}^{-0.62t}+{28.40e}^{-0.22t}$ R^2^ = 1	2.10 ± 3.74 × 10^−1^

VOI = volume of interest.

**Table 5:** Estimates of equivalent and effective radiation doses of the [^99m^Tc]Tc-iPSMA-BN radiopharmaceutical derived from the data obtained from six healthy subjects (three women and three men).

Target Organ	Female Equivalent Dose (mSv/MBq) (Mean ± SD)	Male Equivalent Dose (mSv/MBq) (Mean ± SD)
Adrenals	5.81 × 10^−5^ ± 1.05 × 10^−5^	4.08 × 10^−5^ ± 6.53 × 10^−6^
Brain	8.43 × 10^−6^ ± 1.19 × 10^−6^	8.45 × 10^−6^ ± 1.26 × 10^−6^
Breasts	1.04 × 10^−4^ ± 1.69 × 10^−5^	9.76 × 10^−5^ ± 1.34 × 10^−5^
Gallbladder Wall	4.32 × 10^−5^ ± 9.04 × 10^−6^	3.77 × 10^−5^ ± 5.56 × 10^−6^
LLI Wall	2.32 × 10^−4^ ± 3.21 × 10^−5^	2.14 × 10^−4^ ± 4.99 × 10^−5^
Small Intestine	1.33 × 10^−5^ ± 2.26 × 10^−6^	1.21 × 10^−5^ ± 2.02 × 10^−6^
Stomach Wall	2.71 × 10^−4^ ± 5.23 × 10^−5^	2.49 × 10^−4^ ± 3.52 × 10^−5^
ULI Wall	1.37 × 10^−5^ ± 2.39 × 10^−6^	1.20 × 10^−5^ ± 1.88 × 10^−6^
Heart Wall	2.09 × 10^−5^ ± 3.81 × 10^−6^	1.91 × 10^−5^ ± 2.40 × 10^−6^
Kidneys	4.53 × 10^−4^ ± 1.01 × 10^−4^	3.60 × 10^−4^ ± 8.52 × 10^−5^
Liver	3.21 × 10^−4^ ± 8.00 × 10^−5^	2.49 × 10^−4^ ± 4.10 × 10^−5^
Lungs	1.91 × 10^−4^ ± 2.43 × 10^−5^	1.63 × 10^−4^ ± 2.11 × 10^−5^
Muscle	1.72 × 10^−5^ ± 2.84 × 10^−6^	1.58 × 10^−5^ ± 2.60 × 10^−6^
Ovaries	1.64 × 10^−4^ ± 2.32 × 10^−5^	N.A.
Pancreas	9.84 × 10^−5^ ± 3.58 × 10^−5^	1.23 × 10^−4^ ± 5.15 × 10^−5^
Red Marrow	1.91 × 10^−4^ ± 3.38 × 10^−5^	1.76 × 10^−4^ ± 2.88 × 10^−5^
Osteogenic Cells	3.39 × 10^−5^ ± 6.22 × 10^−6^	3.13 × 10^−5^ ± 5.90 × 10^−6^
Skin	8.06 × 10^−6^ ± 1.28 × 10^−6^	7.83 × 10^−6^ ± 1.22 × 10^−6^
Spleen	4.18 × 10^−5^ ± 8.20 × 10^−6^	3.49 × 10^−5^ ± 5.62 × 10^−6^
Thymus	1.38 × 10^−5^ ± 2.08 × 10^−6^	1.33 × 10^−5^ ± 1.86 × 10^−6^
Thyroid	4.74 × 10^−5^ ± 6.71 × 10^−6^	5.45 × 10^−5^ ± 7.96 × 10^−6^
Urinary Bladder Wall	7.37 × 10^−4^ ± 7.48 × 10^−5^	5.27 × 10^−4^ ± 2.51 × 10^−4^
Uterus	3.10 × 10^−5^ ± 4.17 × 10^−6^	N.A.
Salivary glands	5.58 × 10^−5^ ± 1.50 × 10^−5^	2.82 × 10^−5^ ± 1.19 × 10^−5^
Lacrimal glands	3.47 × 10^−5^ ± 1.50 × 10^−5^	2.57 × 10^−5^ ± 8.76 × 10^−6^
Testes	N.A.	9.43 × 10^−5^ ± 2.24 × 10^−5^
Prostate	N.A.	2.86 × 10^−5^ ± 8.35 × 10^−6^
Effective dose (mSv/MBq)	3.20 × 10^−3^ ± 5.49 × 10^−4^	2.62 × 10^−3^ ± 5.40 × 10^−4^
Effective dose (mSv/740 MBq)	2.37 ± 0.41	1.94 ± 0.39

N.A. = not applicable.

**Table 6:** Analysis of tumor lesions detected with [^18^F]-PSMA-1007 and [^99m^Tc]Tc-iPSMA-BN in patients with metastatic prostate cancer.

Patient No.	[^18^F]–PSMA–1007		[^99m^Tc]Tc–iPSMA–BN	
	Sites of Tumor Lesions (No. Lesions)	TBR (Highest Value)	Sites of Tumor Lesions (No. Lesions)	TBR (Highest Value)
1	P(1), B/R(1), SV(2), LRLN(15), DLN(11)	146.95	P(1), SV(2), LRLN(11), DLN(14)	19
2	P(1), B(>20)	29.50	P(1), B(>20)	39.25
3	P(1), SV(1), B(>20)	61.61	P(1), B(>20)	38.60
4	P(1), B/R(1), SV(2), LRLN(3), DLN(2), B(4)	160	P(1), B/R(1), SV(2), LRLN(3), DLN(3)	41
5	P(1), SV(1), LRLN(6), VD(49)	25.31	P(1), SV(1), LRLN(7), B(2), VD(2)	207
6	P(2), SV(1), LRLN(1), B(17)	13.75	P(1), SV(2), LRLN(1), B(16)	4.52
7	P(2), B/R(1), SV(2), LRLN(1), DLN(4), B(4), VD(1)	37.80	P(1), B/R(1), SV(2), LRLN(1), DLN(3), B(5)	9.90
8	P(1), SV(1), LRLN(4), DLN(3)	20.39	P(1), SV(1), LRLN(5), DLN(1)	78.50
9	P(1), SV(2), LRLN(4), DLN(4)	16.28	P(1), SV(4), LRLN(4), DLN(4)	13.85
10	P(1), LRLN(3), DLN(>20), B(>20)	23.43	P(1), LRLN(3), DLN(>20), B(>20)	24.75
11	P(1), SV(1), DLN(1), B(1)	4.08	P(1), LRLN(1), DLN(3)	5.28

Target-background ratio (TBR), Prostate (P), Seminal vesicles (SV), Bladder and/or rectum (B/R), Locoregional-lymph nodes (LRLN), Distant-lymph nodes (DLN), Bone (B), Visceral disease (VD).

**Table 7:** Estimates of radiation-absorbed doses of [^177^Lu]Lu–iPSMA-BN were derived from the data obtained from healthy subjects (three men).

Target Organ	Absorbed Dose (mGy/MBq) (Mean ± SD)
Adrenals	7.20 × 10^−3^ ± 1.15 × 10^−3^
Brain	4.63 × 10^−3^ ± 6.90 × 10^−4^
Breasts	4.70 × 10^−3^ ± 6.45 × 10^−4^
Gallbladder Wall	7.04 × 10^−3^ ± 1.03 × 10^−3^
LLI Wall	5.20 × 10^−3^ ± 1.21 × 10^−3^
Small Intestine	5.57 × 10^−3^ ± 9.30 × 10^−4^
Stomach Wall	5.70 × 10^−3^ ± 8.06 × 10^−4^
ULI Wall	5.63 × 10^−3^ ± 8.82 × 10^−4^
Heart Wall	5.31 × 10^−3^ ± 6.67 × 10^−4^
Kidneys	6.65 × 10^−1^ ± 1.57 × 10^−1^
Liver	7.00 × 10^−2^ ± 1.15 × 10^−2^
Lungs	5.15 × 10^−3^ ± 6.67 × 10^−4^
Muscle	5.06 × 10^−3^ ± 8.33 × 10^−4^
Pancreas	1.22 × 10^−1^ ± 5.11 × 10^−2^
Red Marrow	4.15 × 10^−3^ ± 6.79 × 10^−4^
Osteogenic Cells	1.51 × 10^−2^ ± 2.85 × 10^−3^
Skin	4.68 × 10^−3^ ± 7.29 × 10^−4^
Spleen	6.54 × 10^−3^ ± 1.06 × 10^−3^
Thymus	4.84 × 10^−3^ ± 6.77 × 10^−4^
Thyroid	4.74 × 10^−3^ ± 6.92 × 10^−4^
Urinary Bladder Wall	9.15 × 10^−2^ ± 4.36 × 10^−2^
Salivary glands	3.93 × 10^−2^ ± 1.19 × 10^−2^
Lacrimal glands	5.50 × 10^−2^ ± 1.83 × 10^−2^
Testes	4.82 × 10^−3^ ± 1.14 × 10^−3^
Prostate	5.55 × 10^−3^ ± 1.62 × 10^−3^
Total body	9.78 × 10^−3^ ± 1.54 × 10^−3^

## Data Availability

Data are contained within this article.
